# Psychosocial stress increases testosterone in patients with borderline personality disorder, post-traumatic stress disorder and healthy participants

**DOI:** 10.1186/s40479-021-00145-x

**Published:** 2021-02-01

**Authors:** Christian E. Deuter, Moritz Duesenberg, Julian Hellmann-Regen, Sophie Metz, Stefan Roepke, Oliver T. Wolf, Christian Otte, Katja Wingenfeld

**Affiliations:** 1 Charité – Universitätsmedizin Berlin, Corporate Member of Freie Universität Berlin, Humboldt-Universität zu Berlin, and Berlin Institute of Health, Klinik für Psychiatrie und Psychotherapie, Campus Benjamin Franklin, 12203 Berlin, Germany; 2 Department of Cognitive Psychology, Institute of Cognitive Neuroscience, Faculty of Psychology, Ruhr University Bochum, Bochum, Germany

**Keywords:** Borderline personality disorder, Post-traumatic stress disorder, Testosterone, Stress response, Psychosocial stress, Trier social stress test

## Abstract

**Background:**

The gonadal hormone testosterone not only regulates sexual behavior but is also involved in social behavior and cognition in both sexes. Changes in testosterone secretion in response to stress have been reported. In addition, stress associated mental disorders such as borderline personality disorder (BPD) and posttraumatic stress disorder (PTSD) are characterized by alterations in basal testosterone metabolism. However, testosterone changes to stress have not been investigated in mental disorders such as BPD and PTSD so far.

**Methods:**

In the study described, we investigated testosterone reactivity to an acute psychosocial stressor, the Trier Social Stress Test (TSST). Our sample consisted of young adult women with BPD (*n* = 28), PTSD (*n* = 22) or both disorders (*n* = 22), and healthy control (*n* = 51). Based on previous studies on basal testosterone secretion in these disorders, we expected the stress-associated testosterone reactivity to be higher in the BPD group and lower in the PTSD group, when compared to the healthy control group.

**Results:**

The study could demonstrate an increase in testosterone after acute stress exposure across all groups and independent of BPD or PTSD status. Different possible explanations for the absence of a group effect are discussed.

**Conclusions:**

From the results of this study, we conclude that stress-related changes in testosterone release are not affected by BPD or PTSD status in a female patient population. This study expands the knowledge about changes in gonadal hormones and stress reactivity in these disorders.

**Supplementary Information:**

The online version contains supplementary material available at 10.1186/s40479-021-00145-x.

## Introduction

Borderline Personality Disorder (BPD) is, among other symptoms, characterized by externalizing behavior, risk taking, antagonism, aggression and impulsivity [29, 34]; behavioral dispositions which have been associated with elevated testosterone levels [17, 28, 52]. In fact, previous studies could demonstrate higher basal testosterone levels in female BPD patients [18, 54, 56], potentially as a consequence of a disturbed androgen metabolism in this patient group [56]. Another mental disorder which is frequently comorbid with BPD is PTSD [51, 64]. Both disorders are associated with traumatic, highly stressful experiences in developmental periods, a factor that has been identified to have lasting effects gonadal hormone regulation [22]. In contrast to BPD, PTSD seems to be associated with reduced testosterone levels: low basal testosterone levels [55] as well as a blunted testosterone reactivity to a CO_2_ inhalation challenge [39] have been shown to promote the development of PTSD, an association with anti-anxiolytic effects of testosterone as well as suppressive effects on HPA activity has been discussed [30]. Testosterone concentrations in cerebrospinal fluid were significantly lower in patients with PTSD when compared to healthy controls [48].

In the current study, we investigated the influence of acute stress on testosterone levels in BPD and PTSD patients, compared to healthy controls. The rational for the study is that both disorders are characterized by abnormal patterns in stress responsivity: symptoms of BPD are oftentimes triggered by stressful situations [9], emotional and behavioral deficits are adversely affected by stress [9, 11, 42, 63]. While divergent response patterns to acute social stress could be demonstrated for cortisol and the autonomic nervous system in BPD [1, 20, 59, 65] and PTSD [46, 62], the testosterone response to acute stress in this patient group has been neglected so far.

While testosterone is secreted in a typical diurnal rhythm. The secretion is also responsive to situational demands [10]. Beyond its primary function in the regulation of sexual behavior, testosterone is in particular relevant in the context of dominance and submission, i.e. in controlling behavior related to hierarchy and social status and dominance behavior [23]. Testosterone levels have been associated with dominance, assertiveness and status seeking; the hormone is released in situations where one’s rank or status is threatened or in situations where status can be gained ([7, 8, 15, 27, 33, 36, 40]; G. A [49].; T [50, 61].). However, testosterone is not only important in negotiating the social hierarchy: it is fundamental for contest and competition in general, e.g. in sports. Elevated testosterone reactivity is related to greater persistence in pursuing goals, greater self-confidence and self-efficacy, as well as greater motivation to perform [24].

Previous studies have investigated the effects of stress on testosterone levels, but the results are equivocal. Testosterone was shown to decrease after prolonged stress exposure [5, 26, 43], but also following acute stress such as the presentation of stressful movie scenes [32] or military survival training [5, 47], anticipatory stress [58] or performance feedback [12]. However, other studies could not corroborate the effect of decreasing testosterone levels after stress exposure [31, 38]. On the other hand and related to its motivation-increasing and anti-anxiolytic effects, the so-called “challenge hypothesis” states that an surge in testosterone levels can be expected in anticipation of a challenge [2]. The main sources of real-life stress in the modern world occur in the social sphere. Social-evaluative stressors are characterized by the fact that they challenge the social self [41]. Reported effects of studies using the TSST, a validated and well-established protocol to induce acute social stress in the laboratory, have also been ambiguous in this regard, with either no change after stress [57] or increased testosterone levels [44], potentially mediated by anxiety, social dominance and status [3, 19, 37, 53]. It has been proposed that initial stages of the stress response could be accompanied by an increase in testosterone release, potentially due to increased sensitivity of gonadal cells to luteinizing hormone; this effect should be more pronounced in dominant individuals and chronic stress [13].

While the research mentioned above allow an estimation of basal testosterone levels in these patient groups, according to our information there are no studies on stress-related testosterone reactivity in these populations. In the present study we investigated testosterone reactivity after acute stress in patients with BPD, PTSD, BPD-PTSD and healthy controls. Stress was induced in the laboratory using the Trier Social Stress Test (TSST), a frequently used and validated stressors in experimental settings. Testosterone in saliva was assessed as a dependent measure. We had different expectations about how both groups of patients react to our manipulation, i.e. what influence stress would have on testosterone reactivity.

In BPD, stress can trigger and increase severity of symptoms [9, 11, 42, 63] and basal testosterone levels are chronically elevated [18, 54, 56]. Taken together, we predicted that in BPD the testosterone level should increase after acute stress. On the other hand, lowered testosterone levels and reactivity to physical stress are associated with PTSD [39, 55]. We thus expected a decrease in testosterone levels after stress induction in this group. These opposite effects should cancel each other out in the group of borderline patients with PTSD. With regard to effects in the healthy control group (HC) we remained agnostic due to the inconsistency in previous research findings. Formally expressed, we expected the following response pattern of testosterone after stress: BPD > [BPD-PTSD = HC] > PTSD.

## Methods

### Participants

The study design was approved by the local ethical committee and in accordance with the latest version of the Declaration of Helsinki. All participants provided written informed consent. Healthy participants and outpatients received monetary compensation (100€) for their participation. We recruited outpatients with BPD, PTSD and healthy participants by public announcements and inpatients from the Department of Psychiatry and Psychotherapy, Campus Benjamin Franklin, Charité Universitätsmedizin Berlin. The described experiment was part of a larger study [21, 46].

Diagnostic criteria for BPD and PTSD were assessed by using the Structured Clinical Interview for DSM-IV axis I and II (SCID) [66]. Healthy participants were excluded if they met diagnostic criteria for any DSM-IV axis I or axis II disorder, reported a history of psychiatric or psychotherapeutic treatment or reported intake of any medication. Any autoimmune or somatic diseases, diseases related to the central nervous system, metabolic or endocrine disorders, infections at the time of study participation, pregnancy, and a body mass index (BMI) below 17.5 and above 30 kg/m^2^ led to exclusion. In addition, healthy participants had no current or previous diagnosis of mental disorder.

We recruited 125 participants, of which two aborted the experiment and had to be excluded. The final dataset consisted of 123 participants: 28 patients with BPD, 22 patients with PTDS, 22 participants with both BPD and PTSD and 51 healthy controls.

### Procedure

Testing sessions were held at 4 pm in the afternoon. We used the Trier Social Stress Test (TSST) as a standardized psychosocial stress test [41] and the Placebo-TSST (P-TSST) as the control condition [35]. Experimental testing took place with at least 1 week between testing sessions, the order of TSST and P-TSST was randomized between participants. Both sessions were conducted within the same menstrual cycle phase (follicular or luteal).

The TSST reliably induces activation of the HPA axis and consists of three phases: preparation, a speech in front of a trained two-person committee and an arithmetic task (5 min each). The speech is based on a job interview: the participant should imagine that she has applied for a self-chosen position and has now been invited to an interview. She should convince the committee that she is the best candidate for the position. Meanwhile, the committee members take notes on the behavior of the “applicant”, a camera and a microphone are supposed to be used for later analysis of speech, facial expressions and gestures. The arithmetic task requires the participant to subtract as quickly and correctly as possible from a four-digit number in steps of 13. The P-TSST is designed to be as similar as possible to the TSST (including orthostatic load) without being stressful to the participant. In an empty room, the participant is asked to talk aloud about a topic of his choice after a preparation phase and to do an easy arithmetic task afterwards (participant is asked to add up the number 15 starting at 0). In an empty room, the participant is asked to talk aloud about a topic of his choice after a preparation phase. As a further part of this study, an experimental memory task was conducted 30 min after TSST onset, with findings being reported elsewhere [21].

### Subjective mood and stress ratings

We used the German version of the ‘Multidimensional Mood State Questionnaire (MDMQ)’ [60] to assess mood immediately before and after the (P) TSST (+ 20 min) and additionally + 80 min after (P) TSST onset. This questionnaire reflects subjective mood state, participants had to rate their current mood on 16 items, related to three dimensions: ‘good vs. bad’, ‘calm vs. nervous’ and ‘awake vs. tired’.

In addition, participants filled out a questionnaires before and after (P) TSST to assess their expectations and experience of the stress manipulation. Specifically, this established 9-item questionnaire [4] asks the participant how challenging, strenuous, controllable, demanding, stressful, new and threatening the situation was expected/experienced, how much they expect to show/have shown a good performance and how important it would be/was for them to show a good performance.

### Sampling and analysis of salivary testosterone

For the determination of salivary testosterone levels, saliva samples were collected using neutral Salivettes® (Blue Cap; Sarstedt, Germany). For sample collection, participants were instructed to move the cotton swab for 1 min around their mouths in a circular pattern to ensure homogenous saliva collection from all salivary glands, without actively chewing on it. Samples were subsequently stored at − 80 °C before further biochemical analyses were performed in the Neurobiology Laboratory of the Dept. of Psychiatry, Charité Universitätsmedizin Berlin.

We sampled saliva at six time points during each testing session: two baseline measurements − 15 min and directly before (P-)TSST, as well as + 20, + 30, + 45 and + 80 min after (P-)TSST onset. Participants were not allowed to drink immediately before collecting salivary samples and were asked not to consume any food, caffeine and sugar containing drinks 1 h before testing.

For the determination of salivary testosterone levels, saliva was recovered from the Salivette® collection system by centrifugation (1000 x g, 2 min., 4 °C). Testosterone levels were determined in 50 μl of saliva using a competitive immunoassay (IBL/TECAN, Hamburg, Germany). The assay procedure followed the manufacturer’s recommendations. The limit of detection was 2 pg /ml, the precision parameters (coefficient of variation; CV) for medium concentrations at 50 pg/ml were determined to average below 5% CV for intra- and 10% CV for inter-assay variance.

### Statistical analysis

SPSS version 22.0 was used for all statistical analyses. Initially we assessed basal testosterone levels for deviating patterns and excluded participants with basal testosterone levels (defined as the averaged pre-TSST values of both testing sessions) above 3 SD from the mean of the total sample (*n* = 16). Because testosterone values were non-normally distributed (Kolmogorov—Smirnov *p* < .05), data were log-transformed. Basal testosterone levels vary considerably between individuals and since we were interested in reactivity, we calculated change scores [3]: for this purpose, the two baseline values were averaged and subtracted from the respective post-intervention values. We used mixed-measures analysis of variance (ANOVA) to investigate the effects of acute stress on testosterone levels, with the between-subjects factor ‘group’ (BPD, PTSD, BPD-PTSD, healthy controls) and the within-subjects factors ‘stress’ (TSST vs. P-TSST) and ‘time’ (+ 20, + 30, + 45, + 80). Testosterone in saliva was measured as a dependent measure. To analyze differences between groups, Bonferroni corrected post-hoc tests were used. Demographic and clinical characteristics were analyzed with one-way ANOVA for continuous variables or chi^2^ test for dichotomous variables across groups. Subjective mood and stress ratings were analyzed by repeated measures ANOVAs, with the between-subjects factor ‘group’ and the within-subjects factors ‘stress’ and ‘time’. For testing of statistical significance, *p*-values smaller than 0.05 were considered to indicate significance. In case of violations of sphericity, reported p-values were Greenhouse-Geisser corrected.

## Results

### Sample characteristics

Groups did not differ with regard to educational status, BMI and the intake of oral contraception. Patients in the PTSD group were significantly older, compared to patients in the BPD-PTSD and BPD groups and healthy controls. Also, there were more smokers in the patient groups than in the healthy controls (see Table 1). In the BPD patient group, 19 patients received psychotropic medication, 1 patient took three and 9 took two different drugs. A total of 9 patients received no psychotropic medication. In the BPD-PTSD patient group, 15 patients received psychotropic medication, 2 patients took three and 5 took two different drugs. A total of 7 patients received no psychotropic medication. In the PTSD patient group, 5 patients took two different drugs. A total of 12 patients received no psychotropic medication. In the healthy control group, no participant received psychotropic medication (see Supplementary Table 1 for details about menstrual cycle phase, comorbid disorders and psychotropic medication).

**Table 1:** Demographic characteristics (mean, standard deviation)

	BPD (*n* = 28)	BPD-PTSD (*n* = 22)	PTSD (*n* = 22)	Healthy Controls (*n* = 51)	Statistics	*p*
Age in years (Mean, SD, Range)	27.54 (6.33) 19–43	29.82 (8.38) 19–47	39.59 (7.73) 24–51	31.69 (9.19) 19–52	*F*(3,119) = 9.53	<.001 *PTSD > BPD=* *BPD-PTSD=HC*
Smoker (n/y)	12/16	9/13	9/13	42/9	*χ*^2^(3) = 20.39	<.001
Years of Education (Mean, SD)	11.61 (1.87)	11.33 (1.74)	11.45 (1.79)	11.69 (1.39)	*F*(3,118) = 0.27	.847
BMI in kg/m^2^ (Mean, SD)	23.51 (3.71)	23.52 (3.09)	24.86 (4.62)	22.53 (2.76)	*F*(3,117) = 2.40	.071
Oral contraception (n/y)	21/7	16/6	18/4	29/22	*χ*^2^(3) = 20.39	.126

Abbr.: *BPD* Borderline Personality Disorder, *PTSD* Post-traumatic stress disorder, *BMI* Body Mass Index

### Subjective mood and stress ratings

#### MDMQ

For all three dimensions of the mood questionnaire, significant effects of stress manipulation were found over time (interaction ‘stress x time’): good/bad (F_2,198_ = 63.30, *p* < .001, η_p_^2^ = .39), calm/nervous (F_2,198_ = 51.87, *p* < .001, η_p_^2^ = .34), and awake/tired (F_2,198_ = 5.26, *p* = .007, η_p_^2^ = .05).

To better understand the exact nature of the interaction effects, Bonferroni-corrected paired samples t-tests were carried out for the P-TSST and the TSST condition. In the placebo condition, mood significantly improved (good vs. bad) directly after P-TSST at + 20 min (*t*_106_ = 3.05, *p* = .003) and at + 80 min (*t*_105_ = 3.23, *p* = .002) when compared to baseline (0 min). In the TSST condition, mood was significantly worse directly after TSST at compared to baseline (*t*_106_ = 8.36, *p* < .001). In the placebo condition, participants felt less nervous directly after P-TSST at + 20 min (*t*_106_ = 3.27, *p* = .001) and at + 80 min (*t*_105_ = 5.08, *p* < .001) when compared to baseline (0 min). In the TSST condition, participants felt more nervous directly after TSST at compared to baseline (*t*_106_ = 8.61, *p* < .001). In the placebo condition, participants felt less nervous directly after P-TSST at + 20 min (*t*_106_ = 3.27, *p* = .001) and at + 80 min (*t*_105_ = 5.08, *p* < .001) when compared to baseline (0 min). In the TSST condition, participants felt more nervous directly after TSST at compared to baseline (*t*_106_ = 8.61, *p* < .001). No individual comparison was significant for the ‘awake vs. tired’ dimension (see Supplement Figure S1a-c).

There were also differences in all three dimensions between the groups (main effect ‘group’): good/bad (F_3,99_ = 25.11, *p* < .001, η_p_^2^ = .43), calm/nervous (F_3,99_ = 17.82, *p* < .001, η_p_^2^ = .35), and awake/tired (F_3,99_ = 17.59, *p* < .001, η_p_^2^ = .35). To better understand the exact nature of these interaction effects, Bonferroni-corrected independent samples t-tests were carried out to compare each group against the others. Across conditions and measurements, the patient groups reported to be in a worse mood (BPD: *t*_66_ = 6.98, *p* < .001, BPD-PTSD: *t*_58_ = 7.88, *p* < .001, PTSD: *t*_63_ = 5.69, *p* < .001), to be more nervous (BPD: *t*_66_ = 6.52, *p* < .001, BPD-PTSD: *t*_58_ = 6.58, *p* < .001, PTSD: *t*_63_ = 5.76, *p* < .001) and more tired (BPD: *t*_66_ = 6.62, *p* < .001, BPD-PTSD: *t*_58_ = 4.23, *p* < .001, PTSD: *t*_63_ = 5.59, *p* < .001) than the healthy controls. However, patient groups did not differ significantly among each other (see Supplement Figure S2).

For the dimension ‘good vs. bad’ a three-way interaction between ‘stress x time x group’ was significant (F_6,198_ = 2.32, *p* = .034, η_p_^2^ = .07): although all groups reported worse mood immediately after the TSST, this was more pronounced in BPD and BPD-PTSD than in the other groups. To better understand the exact nature of this interaction, Bonferroni-corrected paired samples t-tests were carried out for the P-TSST and the TSST condition, separate for each group. The patients in the BPD (*t*_23_ = 7.96, *p* < .001) and BPD-PTSD (*t*_15_ = 6.58, *p* < .001) group as well as the healthy controls (*t*_45_ = 5.62, *p* < .001) reported significantly worse mood in the stress condition immediately after the TSST (+ 20 min) when compared to baseline (see Supplement Figure S3).

#### Subjective stress questionnaire

In the subjective stress questionnaire, we found significant interactions ‘stress x time’ for the following items: participants experienced the TSST as more challenging (F_1,103_ = 77.96, *p* < .001, η_p_^2^ = .43), strenuous (F_1,103_ = 129.48, *p* < .001, η_p_^2^ = .58), demanding (F_1,103_ = 119.11, *p* < .001, η_p_^2^ = .54), stressful (F_1,103_ = 139.01, *p* < .001, η_p_^2^ = .57) and threatening (F_1,103_ = 124.68, *p* < .001, η_p_^2^ = .55), less controllable (F_1,103_ = 112.68, *p* < .001, η_p_^2^ = .52) and had less confidence in their performance (F_1,103_ = 102.30, *p* < .001, η_p_^2^ = .50) when compared to the non-stressful P-TSST.

We also found an interaction ‘stress x time x group’ for the question “I expect the situation to be threatening” and “I experienced the situation as threatening” (F_3,103_ = 6.44, *p* < .001, η_p_^2^ = .16): while all groups experienced the TSST as more threatening than expected when compared to the P-TSST, this effect was more pronounced for the patient groups (BPD, BPD-PTSD and PTSD). To better understand the exact nature of this interaction, Bonferroni-corrected independent samples t-tests were carried out between groups, separate for the P-TSST and the TSST condition. The patient groups prior to P-TSST indicated a higher expectation that the situation would become threatening than the control group (BPD: *t*_68_ = 5.06, *p* < .001, BPD-PTSD: *t*_60_ = 4.59, *p* < .001, PTSD: *t*_65_ = 2.88, *p* = .005), but did not differ among each other. After the TSST, the patient groups agreed more strongly with the statement that the situation was threatening than the control group (BPD: *t*_68_ = 3.36, *p* = .001, BPD-PTSD: *t*_60_ = 3.59, *p* = .001, PTSD: *t*_65_ = 3.71, *p* < .001), but again did not differ among each other (see Supplement Figure S4).

### Testosterone response to stress

**Fig. 1:**
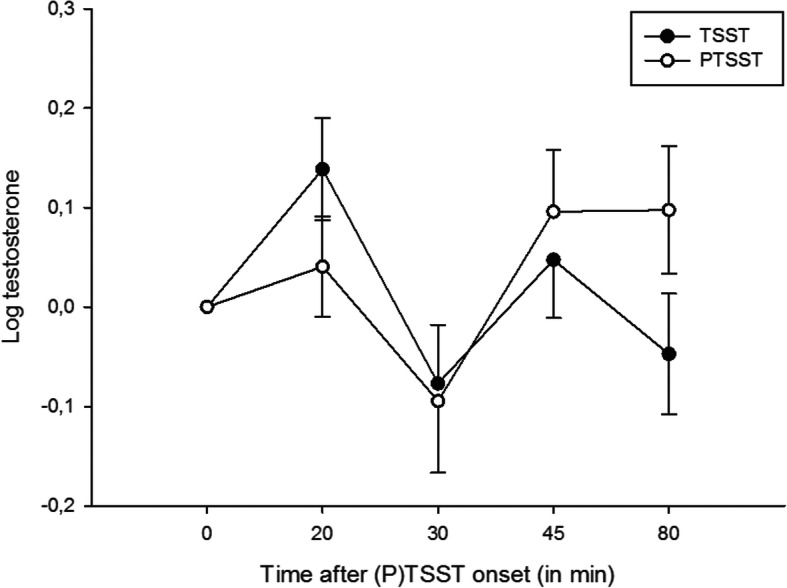
Testosterone reactivity after stress manipulation (TSST) and non-stressful placebo intervention (P-TSST). Data points represent change scores (testosterone score at time of measurement minus the average of two baseline samples). Testosterone values were log-transformed. Post-hoc tests showed a significant increase in testosterone levels + 20 min after TSST compared to the baseline. Error bars represent ±1 SEM

We found a significant main effect of ‘time’ (F_3,273_ = 4.69, *p* = .005, η_p_^2^ = .05), indicating changes in testosterone levels across measurements, and a significant interaction between ‘stress x time’ (F_3,261_ = 3.46, *p* = .02, η_p_^2^ = .04), which suggests that this change was affected by the stress manipulation (see Fig. 1, see Supplement Table S2 for raw values and Supplement Figure S5 for group specific results). The main effect of ‘stress’ (F_1,261_ = 0.38, *p* = .54, η_p_^2^ = .004) was not significant.

To better understand the exact nature of the interaction effect, we calculated separate repeated measures ANOVAs for TSST and P-TSST. The effect of ‘time’ was statistically significant for the TSST condition (F_3,297_ = 7.81, *p* < .001, η_p_^2^ = .073), which was not the case in the placebo condition (P-TSST: F_3,294_ = 2.82, *p* = .044, η_p_^2^ = .028) after adjusting for multiple testing. Subsequently, we calculated paired-sample *t*-tests, testing the change from the baseline for each individual measurement point. After Bonferroni correction there was a significant difference only for the change from baseline at the first measurement time after + 20 min in the TSST condition (*t*_100_ = 2.64, *p* = .010): testosterone levels increased from baseline after the TSST.

None of the effects involving the factor ‘group’ turned out to be significant: neither BPD nor PTSD had a moderating effect on testosterone reactivity after stress (‘group’: F_1,91_ = 1.06, *p* = .37, η_p_^2^ = .034, ‘group x stress’: F_3,91_ = 1.34, *p* = .27, η_p_^2^ = .042, ‘group x time’: F_9,273_ = 0.53, *p* = .83, η_p_^2^ = .017, ‘group x stress x time’: F_9,273_ = 0.94, *p* = .49, η_p_^2^ = .03).

### Correlations between testosterone response and subjective measures

In an exploratory analysis, we investigated the correlations between the peak testosterone responses in the TSST condition (change score at + 20 min) and the three dimensions of the MDMQ as well as the nine items of the subjective stress questionnaire. None of those correlations was significant, with all *p* > .05.

## Discussion

The aim of this study was to investigate the influence of different psychiatric disorders, specifically BPD and PTSD, on testosterone reactivity after stress. We assumed that the testosterone response would be different in both disorders and would differ from a healthy population. We could not confirm our primary hypothesis: while testosterone increased across groups after stress, we found no between-group differences in testosterone reactivity to stress.

In the stress condition (TSST), participants exhibited increased testosterone reactivity compared to the non-stress control condition (P-TSST). We were thus able to replicate previous studies [3, 19, 44, 53]. The increase in testosterone levels after TSST corresponds to elevated testosterone levels during competition and could be an indication that the participants regarded the psychosocial stress situation as a personal challenge. The TSST with an authoritarian committee and a threat to the participants’ social self corresponds to a situation in which dominance and submission are negotiated, and that tend to increase testosterone secretion. Besides that, the anabolic functions of testosterone could exert a protective and regenerative role against potential negative consequences of stress [44]. Nevertheless, this effect is in contrast to other previous studies that found no effect [31, 57] or a reduction in testosterone after stress [32, 38]. We can only speculate about the causes of the deviating findings. A major difference is the composition of our sample. In comparison to the latter studies, our sample was comparatively large, consisted mainly of patients with mental disorders and exclusively of female participants. Compared to the healthy control group, patient groups in our sample experienced worse mood after the TSST and felt more threatened by the situation. Even if no statistically significant group differences in testosterone reactivity were found, our clinical sample may have been more susceptible to the stress manipulation than the average healthy population. Furthermore, it has been argued that the testosterone production in men and women follows different metabolic pathways and is primarily of adrenal origin in women – which could make testosterone secretion in women more responsive to stress [27, 53].

The main aim of the study was to investigate disorder-specific patterns in the stress response of testosterone. For BPD as well as for PTSD this relationship was not investigated so far. Also for other psychiatric disorders there is hardly any data available, the only study known to us was conducted in male adolescents with anxiety disorder and found an increase of testosterone after stress in patients but not in the control group [25]. Since such disorder-specific response patterns have been shown for other biological stress systems in BPD and PTSD, this assumption also seemed justified for testosterone. Assuming that anger, aggressiveness, and antagonizing behavior characterize BPD and that stress has a contribution to BPD symptoms, we hypothesized to find an increased release of testosterone after stress in this group. This increased secretion would have provided an explanatory model to mechanistically explain this aspect of BPD. Furthermore and based on the existing literature, we expected to find the opposite pattern for BPD. However, in our study there were no differences between groups in this respect. What could be potential causes? As stated in the introduction, studies on the influence of stress on testosterone reactivity in healthy participants have shown heterogeneous results. While more recent studies using the TSST found either no effects or an increase in testosterone, those effects were comparatively small. No such studies have previously been conducted in patients with BPD and PTSD. Our sample was rather large (*n* > 100) and given an error probability of 1-β = 0.9, statistical power was sufficient to detect small to medium sized effects of η2 = 0.2 or higher. Statistical power might not have been sufficient to detect smaller between-group effects. Initially we speculated that the two disorders are characterized by opposing response patterns. Thus, these potentially existing effects may have mutually neutralized each other in total in our study, without becoming statistically significant as a between group effect. However, the statistical parameters for effects involving the factor ‘group’ were very small and no descriptive differences were apparent. We therefore have reason to believe that these effects do exist in the population we were investigating. Another possible explanation is the composition of our sample: we conducted the study only on women. Although the vast majority of PTSD patients are women [14], the associations between PTSD and testosterone described in the existing literature have, to the best of our knowledge, only been investigated in men and in a military context. For BPD, there is also evidence of altered testosterone metabolism in female patients [18, 54]. However, gender differences exist for BPD in different domains such as impulsivity or aggressiveness, which could be at least partly related to underlying neurobiological and endocrinal differences [6, 45, 54]. Furthermore, very recent findings suggest that the relationship between testosterone on the one hand and anger/aggression on the other differs between men and women [16]: the experience of stressful life events (assessed by the ‘Stressful Life Events Screening Questionnaire’, SLESQ) and higher levels of testosterone in relation to cortisol reactivity were positively associated with anger and antisocial behavior in (healthy) male participants, but not in female participants. Taken together, there are indications that the association between psychiatric disorders on the one hand and stress/testosterone on the other could be different in men and women.

## Conclusions

This study is, to our knowledge, the first to investigate this subject in BPD and PTSD patients. It thus represents an important extension to studies that have investigated the effect of stress on other physiological parameters such as sympathetic nervous system activity and cortisol reactivity. We could demonstrate a stress responsivity of testosterone secretion, with increased levels after acute stress exposure. Yet, we could not confirm our main hypothesis of group-level differences with regard to stress induced testosterone release. The study aimed to provide a biological explanatory model for certain symptoms of BPD, from which therapeutic options could have potentially emerged. Future studies could examine subgroups of BPD and stratify by behavioral response patterns to stress, i.e. patients more prone to anger and aggressiveness vs. more “passive” coping strategies such as dissociation. Furthermore, we have only investigated the relationships within a female only sample. This is representative insofar as both disorders are mostly found in women. However, there are indications that the relationships examined are different in men. Future studies should also take these gender differences into account.

## Supplementary Information

**Additional file 1: Figure S1a**. MDMQ subjective mood ratings: good vs. bad (mean +/- 1SE). **Figure S1b.** MDMQ subjective mood ratings: calm vs. nervous (mean +/- 1SE). **Figure S1c.** MDMQ subjective mood ratings: awake vs. tired (mean +/- 1SE). **Figure S2**. MDMQ mood ratings by group, across time and condition (mean +/- 1SE). **Figure S3.** MDMQ mood ratings by group, time and condition (mean +/- 1SE). **Figure S4.** Subjective stress questionnaire ratings by group, time and condition (mean +/− 1SE). **Figure S5.** Testosterone reactivity after stress manipulation (TSST) and non-stressful placebo intervention (PTSST), separate lines for each group. Data points represent change scores (testosterone scor e at time of measurement minus the average of two baseline samples). Testosterone values were log-transformed. (mean +/− 1SE). **Table S1.** Description of the sample with respect to menstrual cycle phase (only for participants without hormonal contraception), Comorbidcurrent DSM-IV axis diagnoses and psychotropic medication, in number of participants, Abbr: BPD = borderline personality disorder, PTSD = post-traumatic stress disorder, SSRI = selective serotonin reuptake inhibitor, SNRI = serotonin and noradrenaline reuptake inhibitor, NDRI = dopamine and noradrenergic reuptake inhibitor. **Table S2.** Raw testosterone values in pg/ml, means and standard deviation.

## Data Availability

The datasets analysed during the current study are available from the corresponding author on reasonable request.

## References

[1] Aleknaviciute J, Tulen JH, Kamperman AM, de Rijke YB, Kooiman CG, Kushner SA (2016). Borderline and cluster C personality disorders manifest distinct physiological responses to psychosocial stress. Psychoneuroendocrinology.

[2] Archer J (2006). Testosterone and human aggression: an evaluation of the challenge hypothesis. Neurosci Biobehav Rev.

[3] Bedgood D, Boggiano MM, Turan B (2014). Testosterone and social evaluative stress: the moderating role of basal cortisol. Psychoneuroendocrinology.

[4] Bellingrath S, Kudielka BM (2008). Effort-reward-imbalance and overcommitment are associated with hypothalamus-pituitary-adrenal (HPA) axis responses to acute psychosocial stress in healthy working schoolteachers. Psychoneuroendocrinology.

[5] Bernton E, Hoover D, Galloway R, Popp K (1995). Adaptation to chronic stress in military trainees. Adrenal androgens, testosterone, glucocorticoids, IGF-1, and immune function. Ann N Y Acad Sci.

[6] Bertsch K, Roelofs K, Roch PJ, Ma B, Hensel S, Herpertz SC (2018). Neural correlates of emotional action control in anger-prone women with borderline personality disorder. J Psychiatry Neurosci.

[7] Booth A, Granger DA, Mazur A, Kivlighan K (2006). Testosterone and social behavior. Social Forces.

[8] Bos PA, van Honk J, Ramsey NF, Stein DJ, Hermans EJ (2013). Testosterone administration in women increases amygdala responses to fearful and happy faces. Psychoneuroendocrinology.

[9] Bourvis N, Aouidad A, Cabelguen C, Cohen D, Xavier J (2017). How do stress exposure and stress regulation relate to borderline personality disorder?. Front Psychol.

[10] Brambilla DJ, Matsumoto AM, Araujo AB, McKinlay JB (2009). The effect of diurnal variation on clinical measurement of serum testosterone and other sex hormone levels in men. J Clin Endocrinol Metab.

[11] Cackowski S, Krause-Utz A, Van Eijk J, Klohr K, Daffner S, Sobanski E (2017). Anger and aggression in borderline personality disorder and attention deficit hyperactivity disorder - does stress matter?. Borderline Personal Disord Emot Dysregul.

[12] Campbell J, Fiacco S, Ditzen B, Meuwly N, Ehlert U (2019). Endocrine correlates of social comparison in couple relationships. Adapt Hum Behav Physiol.

[13] Chichinadze K, Chichinadze N (2008). Stress-induced increase of testosterone: contributions of social status and sympathetic reactivity. Physiol Behav.

[14] Christiansen DM, Berke ET (2020). Gender- and sex-based contributors to sex differences in PTSD. Curr Psychiatry Rep.

[15] Cook CJ, Crewther BT, Smith AA (2012). Comparison of baseline free testosterone and cortisol concentrations between elite and non-elite female athletes. Am J Hum Biol.

[16] Cooke EM, Connolly EJ, Boisvert DL, Armstrong TA, Lewis RH, Kavish N, et al. Examining how testosterone and cortisol influence the relationship between strain, negative emotions, and antisocial behavior: a gendered analysis. Crime Delinq. 2020. 10.1177/0011128720903047.

[17] Dabbs JM, Hargrove MF (1997). Age, testosterone, and behavior among female prison inmates. Psychosom Med.

[18] Dettenborn L, Kirschbaum C, Gao W, Spitzer C, Roepke S, Otte C (2016). Increased hair testosterone but unaltered hair cortisol in female patients with borderline personality disorder. Psychoneuroendocrinology.

[19] Deuter CE, Schachinger H, Best D, Neumann R (2016). Effects of two dominance manipulations on the stress response: cognitive and embodied influences. Biol Psychol.

[20] Drews E, Fertuck EA, Koenig J, Kaess M, Arntz A (2019). Hypothalamic-pituitary-adrenal axis functioning in borderline personality disorder: a meta-analysis. Neurosci Biobehav Rev.

[21] Duesenberg M, Wolf OT, Metz S, Roepke S, Fleischer J, Elias V (2019). Psychophysiological stress response and memory in borderline personality disorder. Eur J Psychotraumatol.

[22] Eck SR, Bangasser DA (2020). The effects of early life stress on motivated behaviors: a role for gonadal hormones. Neurosci Biobehav Rev.

[23] Eisenegger C, Haushofer J, Fehr E (2011). The role of testosterone in social interaction. Trends Cogn Sci.

[24] Eisenegger C, Naef M, Snozzi R, Heinrichs M, Fehr E (2010). Prejudice and truth about the effect of testosterone on human bargaining behaviour. Nature.

[25] Gerra G, Zaimovic A, Zambelli U, Timpano M, Reali N, Bernasconi S (2000). Neuroendocrine responses to psychological stress in adolescents with anxiety disorder. Neuropsychobiology.

[26] Gomez-Merino D, Drogou C, Chennaoui M, Tiollier E, Mathieu J, Guezennec CY (2005). Effects of combined stress during intense training on cellular immunity, hormones and respiratory infections. Neuroimmunomodulation.

[27] Grant VJ, France JT (2001). Dominance and testosterone in women. Biol Psychol.

[28] Grotzinger AD, Mann FD, Patterson MW, Tackett JL, Tucker-Drob EM, Harden KP (2018). Hair and salivary testosterone, hair cortisol, and externalizing behaviors in adolescents. Psychol Sci.

[29] Gunderson JG, Herpertz SC, Skodol AE, Torgersen S, Zanarini MC (2018). Borderline personality disorder. Nat Rev Dis Primers.

[30] Handa RJ, Weiser MJ (2014). Gonadal steroid hormones and the hypothalamo-pituitary-adrenal axis. Front Neuroendocrinol.

[31] Heinz A, Hermann D, Smolka MN, Rieks M, Gräf KJ, Pöhlau D (2003). Effects of acute psychological stress on adhesion molecules, interleukins and sex hormones: implications for coronary heart disease. Psychopharmacology.

[32] Hellhammer DH, Hubert W, Schurmeyer T (1985). Changes in saliva testosterone after psychological stimulation in men. Psychoneuroendocrinology.

[33] Henry A, Sattizahn JR, Norman GJ, Beilock SL, Maestripieri D (2017). Performance during competition and competition outcome in relation to testosterone and cortisol among women. Horm Behav.

[34] Herpertz SC, Nagy K, Ueltzhöffer K, Schmitt R, Mancke F, Schmahl C (2017). Brain mechanisms underlying reactive aggression in borderline personality disorder-sex matters. Biol Psychiatry.

[35] Het S, Rohleder N, Schoofs D, Kirschbaum C, Wolf OT (2009). Neuroendocrine and psychometric evaluation of a placebo version of the ‘Trier social stress test’. Psychoneuroendocrinology.

[36] Jimenez M, Aguilar R, Alvero-Cruz JR (2012). Effects of victory and defeat on testosterone and cortisol response to competition: evidence for same response patterns in men and women. Psychoneuroendocrinology.

[37] Jin ES, Josephs RA (2017). Greater testosterone reactivity associated with lower subjective anxiety in response to social stressor. Psychoneuroendocrinology.

[38] Johansson GG, Laakso ML, Peder M, Karonen SL (1988). Examination stress decreases plasma level of luteinizing hormone in male students. Psychosom Med.

[39] Josephs RA, Cobb AR, Lancaster CL, Lee HJ, Telch MJ (2017). Dual-hormone stress reactivity predicts downstream war-zone stress-evoked PTSD. Psychoneuroendocrinology.

[40] Josephs RA, Mehta PH, Carré JM (2011). Gender and social environment modulate the effects of testosterone on social behavior: comment on Eisenegger et al. Trends Cogn Sci.

[41] Kirschbaum C, Pirke K-M, Hellhammer DH (1993). The ‘Trier social stress test’–a tool for investigating psychobiological stress responses in a laboratory setting. Neuropsychobiology.

[42] Krause-Utz A, Cackowski S, Daffner S, Sobanski E, Plichta MM, Bohus M (2016). Delay discounting and response disinhibition under acute experimental stress in women with borderline personality disorder and adult attention deficit hyperactivity disorder. Psychol Med.

[43] Kreuz LE, Rose RM, Jennings JR (1972). Suppression of plasma testosterone levels and psychological stress. A longitudinal study of young men in officer candidate school. Arch Gen Psychiatry.

[44] Lennartsson AK, Kushnir MM, Bergquist J, Billig H, Jonsdottir IH (2012). Sex steroid levels temporarily increase in response to acute psychosocial stress in healthy men and women. Int J Psychophysiol.

[45] Mancke F, Bertsch K, Herpertz SC (2015). Gender differences in aggression of borderline personality disorder. Borderline Personal Disord Emot Dysregul.

[46] Metz S, Duesenberg M, Hellmann-Regen J, Wolf OT, Roepke S, Otte C (2020). Blunted salivary cortisol response to psychosocial stress in women with posttraumatic stress disorder. J Psychiatr Res.

[47] Morgan CA, Wang S, Mason J, Southwick SM, Fox P, Hazlett G (2000). Hormone profiles in humans experiencing military survival training. Biol Psychiatry.

[48] Mulchahey JJ, Ekhator NN, Zhang H, Kasckow JW, Baker DG, Geracioti TD (2001). Cerebrospinal fluid and plasma testosterone levels in post-traumatic stress disorder and tobacco dependence. Psychoneuroendocrinology.

[49] Oliveira GA, Uceda S, Oliveira T, Fernandes A, Garcia-Marques T, Oliveira RF (2013). Threat perception and familiarity moderate the androgen response to competition in women. Front Psychol.

[50] Oliveira T, Gouveia MJ, Oliveira RF (2009). Testosterone responsiveness to winning and losing experiences in female soccer players. Psychoneuroendocrinology.

[51] Pagura J, Stein MB, Bolton JM, Cox BJ, Grant B, Sareen J (2010). Comorbidity of borderline personality disorder and posttraumatic stress disorder in the U.S. population. J Psychiatr Res.

[52] Pajer K, Tabbah R, Gardner W, Rubin RT, Czambel RK, Wang Y (2006). Adrenal androgen and gonadal hormone levels in adolescent girls with conduct disorder. Psychoneuroendocrinology.

[53] Phan JM, Schneider E, Peres J, Miocevic O, Meyer V, Shirtcliff EA (2017). Social evaluative threat with verbal performance feedback alters neuroendocrine response to stress. Horm Behav.

[54] Rausch J, Gäbel A, Nagy K, Kleindienst N, Herpertz SC, Bertsch K (2015). Increased testosterone levels and cortisol awakening responses in patients with borderline personality disorder: gender and trait aggressiveness matter. Psychoneuroendocrinology.

[55] Reijnen A, Geuze E, Vermetten E (2015). The effect of deployment to a combat zone on testosterone levels and the association with the development of posttraumatic stress symptoms: a longitudinal prospective Dutch military cohort study. Psychoneuroendocrinology.

[56] Roepke S, Ziegenhorn A, Kronsbein J, Merkl A, Bahri S, Lange J (2010). Incidence of polycystic ovaries and androgen serum levels in women with borderline personality disorder. J Psychiatr Res.

[57] Schoofs D, Wolf OT (2011). Are salivary gonadal steroid concentrations influenced by acute psychosocial stress? A study using the Trier social stress test (TSST). Int J Psychophysiol.

[58] Schulz P, Walker JP, Peyrin L, Soulier V, Curtin F, Steimer T (1996). Lower sex hormones in men during anticipatory stress. Neuroreport.

[59] Simeon D, Knutelska M, Smith L, Baker BR, Hollander E (2007). A preliminary study of cortisol and norepinephrine reactivity to psychosocial stress in borderline personality disorder with high and low dissociation. Psychiatry Res.

[60] Steyer R, Schwenkmezger P, Notz P, Eid M. Testtheoretische Analysen des Mehrdimensionalen Befindlichkeitsfragebogen (MDBF). Diagnostica. 1994.

[61] van Honk J, Tuiten A, Hermans E, Putman P, Koppeschaar H, Thijssen J (2001). A single administration of testosterone induces cardiac accelerative responses to angry faces in healthy young women. Behav Neurosci.

[62] Wichmann S, Kirschbaum C, Böhme C, Petrowski K (2017). Cortisol stress response in post-traumatic stress disorder, panic disorder, and major depressive disorder patients. Psychoneuroendocrinology.

[63] Wingenfeld K, Duesenberg M, Fleischer J, Roepke S, Dziobek I, Otte C (2018). Psychosocial stress differentially affects emotional empathy in women with borderline personality disorder and healthy controls. Acta Psychiatr Scand.

[64] Wingenfeld K, Mensebach C, Rullkoetter N, Schlosser N, Schaffrath C, Woermann FG (2009). Attentional bias to personally relevant words in borderline personality disorder is strongly related to comorbid posttraumatic stress disorder. J Personal Disord.

[65] Wingenfeld K, Spitzer C, Rullkötter N, Löwe B (2010). Borderline personality disorder: hypothalamus pituitary adrenal axis and findings from neuroimaging studies. Psychoneuroendocrinology.

[66] Wittchen H-U, Zaudig M, Fydrich T (1997). Skid. Strukturiertes klinisches interview für DSM-IV. Achse I und II. Handanweisung.

