# Supplementary material for: Psychosocial stress increases testosterone in patients with borderline personality disorder, post-traumatic stress disorder and healthy participants
Source: Borderline Personal Disord Emot Dysregul. 2021 Feb 1;8:3. doi: 10.1186/s40479-021-00145-x (PMC7849084; doi:10.1186/s40479-021-00145-x)
Supplement: Supplementary file 1 — Additional file 1: Figure S1a. MDMQ subjective mood ratings: good vs. bad (mean +/- 1SE). Figure S1b. MDMQ subjective mood ratings: calm vs. nervous (mean +/- 1SE). Figure S1c. MDMQ subjective mood ratings: awake vs. tired (mean +/- 1SE). Figure S2. MDMQ mood ratings by group, across time and condition (mean +/- 1SE). Figure S3. MDMQ mood ratings by group, time and condition (mean +/- 1SE). Figure S4. Subjective stress questionnaire ratings by group, time and condition (mean +/− 1SE). Figure S5. Testosterone reactivity after stress manipulation (TSST) and non-stressful placebo intervention (PTSST), separate lines for each group. Data points represent change scores (testosterone scor e at time of measurement minus the average of two baseline samples). Testosterone values were log-transformed. (mean +/− 1SE). Table S1. Description of the sample with respect to menstrual cycle phase (only for participants without hormonal contraception), Comorbidcurrent DSM-IV axis diagnoses and psychotropic medication, in number of participants, Abbr: BPD = borderline personality disorder, PTSD = post-traumatic stress disorder, SSRI = selective serotonin reuptake inhibitor, SNRI = serotonin and noradrenaline reuptake inhibitor, NDRI = dopamine and noradrenergic reuptake inhibitor. Table S2. Raw testosterone values in pg/ml, means and standard deviation. [file 40479_2021_145_MOESM1_ESM.docx]

Fig.1a: MDMQ subjective mood ratings: good vs. bad (mean +/- 1SE)

Fig.1b: MDMQ subjective mood ratings: calm vs. nervous (mean +/- 1SE)

Fig.1c: MDMQ subjective mood ratings: awake vs. tired (mean +/- 1SE)

Fig.2: MDMQ mood ratings by group, across time and condition (mean +/- 1SE)

Fig.3: MDMQ mood ratings by group, time and condition (mean +/- 1SE)

Fig.4: Subjective stress questionnaire ratings by group, time and condition (mean +/- 1SE)


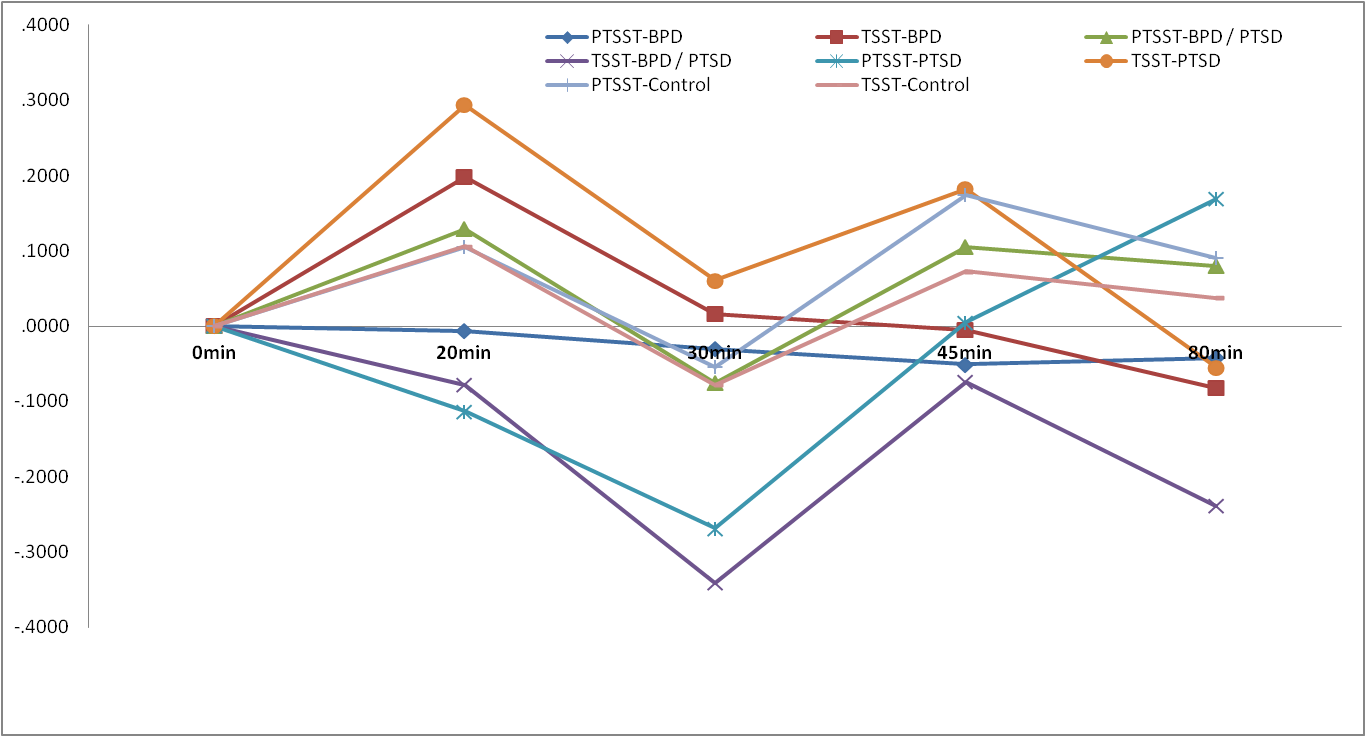


*Supplementary Fig.5: Testosterone reactivity after stress manipulation (TSST) and non-stressful placebo intervention (PTSST), separate lines for each group. Data points represent change scores (testosterone scor e at time of measurement minus the average of two baseline samples). Testosterone values were log-transformed. (mean +/- 1SE)*

|  | BPD | BPD+PTSD | PTSD | Healthy Controls | |
| --- | --- | --- | --- | --- | --- |
| Menstrual cycle phase  follicular  luteal  postmenopausal  n.a. | 3  13  -  3 | 1  10  -  5 | 5  6  1  5 | | 7  14  1  4 |
| Comorbid current  DSM-IV axis diagnoses  agoraphobia  agoraphobia with panic disorder  bulimia nervosa  dissociative disorder  generalized anxiety disorder  obsessive compulsive disorder  panic disorder  social phobia  somatoform disorder  specific phobia  substance abuse | -  -  1  -  1  2  -  3  -  -  1 | -  4  -  1  2  1  3  1  1  1 | 2  1  1  -  -  4  3  2  -  -  2 | | -  -  -  -  -  -  -  -  -  -  - |
| Psychotropic medication  SSRI  SNRI  tricyclic antidepressant  tetracyclic antidepressant  NDRI  antipsychotics  anticonvulsants  alpha/beta adrenergic blocker | 16  2  2  2  1  3  4  1 | 7  2  1  1  1  7  3  2 | 3  4  2  1  2  2  -  1 | | -  -  -  -  -  -  -  - |

***Supplementary Table 1:*** *Description of the sample with respect to menstrual cycle phase (only for participants without hormonal contraception), Comorbidcurrent DSM-IV axis diagnoses and psychotropic medication, in number of participants, Abbr: BPD=borderline personality disorder, PTSD=post-traumatic stress disorder, SSRI = selective serotonin reuptake inhibitor, SNRI= serotonin and noradrenaline reuptake inhibitor, NDRI =dopamine and noradrenergic reuptake inhibitor*

|  | | P-TSST 15min | P-TSST 0min | P-TSST 20min | P-TSST 30min | P-TSST 45min | P-TSST 80min | TSST -15min | TSST 0min | TSST 20min | TSST 30min | TSST 45min | TSST 80min |
| --- | --- | --- | --- | --- | --- | --- | --- | --- | --- | --- | --- | --- | --- |
|  |  |  |  |  |  |  |  |  |  |  |  |  |  |
| BPD | Mean | 29.16 | 29.92 | 25.91 | 24.96 | 26.36 | 27.45 | 28.59 | 28.98 | 35.11 | 29.39 | 28.04 | 26.98 |
|  | SD | 26.59 | 29.99 | 15.19 | 13.28 | 16.90 | 19.18 | 18.11 | 19.18 | 22.03 | 18.89 | 14.95 | 14.04 |
| BPD / PTSD | Mean | 19.23 | 17.97 | 20.42 | 16.82 | 20.47 | 20.32 | 36.75 | 38.62 | 37.28 | 31.45 | 37.96 | 34.29 |
|  | SD | 11.23 | 10.34 | 10.62 | 9.03 | 10.79 | 10.53 | 27.09 | 46.96 | 36.05 | 37.32 | 36.33 | 33.70 |
| PTSD | Mean | 16.98 | 17.65 | 17.67 | 16.30 | 18.54 | 26.23 | 23.16 | 19.65 | 28.41 | 23.26 | 25.58 | 21.64 |
|  | SD | 8.43 | 11.27 | 13.82 | 9.61 | 12.66 | 30.01 | 16.06 | 12.18 | 24.91 | 12.48 | 13.85 | 15.57 |
| Control | Mean | 23.37 | 21.89 | 26.38 | 22.80 | 27.63 | 28.35 | 51.04 | 44.12 | 48.28 | 45.86 | 51.57 | 49.16 |
|  | SD | 19.38 | 18.96 | 24.05 | 21.28 | 22.10 | 25.73 | 54.59 | 51.27 | 44.46 | 48.98 | 48.59 | 47.54 |
| Total | Mean | 22.80 | 22.27 | 23.71 | 21.13 | 24.49 | 26.54 | 38.39 | 35.10 | 39.94 | 35.69 | 39.42 | 37.19 |
|  | SD | 19.03 | 20.18 | 19.05 | 16.54 | 18.18 | 23.47 | 40.33 | 40.36 | 36.35 | 37.69 | 37.75 | 37.28 |

***Supplementary Table 2****: Raw testosterone values in pg/ml, means and standard deviation*
